# The Gut–Brain–Immune Axis: Multi-Omics Insights into Neurodegenerative and Metabolic Diseases

**DOI:** 10.3390/cells15121089

**Published:** 2026-06-16

**Authors:** Salah-Ud-Din Khan, Varun Chauhan, Anis Ahmad Chaudhary, Mohsin Khan

**Affiliations:** 1 Department of Biochemistry, College of Medicine, Imam Mohammad Ibn Saud Islamic University (IMSIU), Riyadh 11623, Saudi Arabia; 2 Multidisciplinary Research Unit, Government Institute of Medical Sciences, Greater Noida 201310, India; 3 Department of Biology, College of Science, Imam Mohammad Ibn Saud Islamic University (IMSIU), Riyadh 11623, Saudi Arabia; 4 Department of Education, Government of Jharkhand, Ranchi 834006, India

**Keywords:** dysbiosis, gut–brain–immune axis, metabolic diseases, metabolomics, multi-omics, neurodegenerative diseases

## Abstract

**Highlights:**

**What are the main findings?**

- Gut microbiota dysbiosis, chronic inflammation, microbial metabolites, and barrier dysfunction are strongly associated with neurodegenerative and metabolic diseases through the gut–brain–immune axis.
- Multi-omics technologies and AI-assisted analyses provide integrated insights into shared molecular pathways, biomarker discovery, and disease mechanisms.

**What is the implication of the main finding?**

- Systems-level integration of microbiome, immune, metabolic, and omics data may support future precision medicine approaches for neurological and metabolic disorders.
- Microbiome-targeted therapies including probiotics, precision nutrition, metabolite-based interventions, and AI-guided strategies remain promising but require further large-scale clinical validation.

**Abstract:**

The axis linking the gut to the brain to the immune system connects all tissues involved—bacteria, immune cells, metabolism and the CNS—through a multidirectional communication network. Several studies have confirmed that when this axis is disrupted, it can be responsible for Alzheimer’s disease, Parkinson’s disease, obesity, type 2 diabetes, and NAFLD, and the main consequences come from increased systemic inflammation, altered regulation of immune cells, the production of microbial metabolites that alter signals to the immune cells and nervous system, increase in oxidative stress, breakdown of the gut barrier, and more. In recent years, advanced multi-omics technologies, such as metagenomics, transcriptomics, metabolomics, proteomics, and single-cell sequencing, have provided significant advancement in our understanding of all of the interacting nodes involved in the gut–brain–immune axis. These advanced sequencing technologies can characterize the microbial communities, host immune cells, metabolic profiles, and the degree of cell heterogeneity during a specific disease. Combining multi-omics information can reveal a few shared pathways between neurodegenerative and metabolic disorders, such as NF-κB, NLRP3 inflammasome activation, mitochondrial dysfunction, changes in SCFA metabolism, and the alteration of microbial populations in Alzheimer’s and Parkinson’s disease; metabolic dysbiosis and increased risk for Parkinson’s disease; or changes in gut-to-brain-to-immune signaling contributing to diabetes complications and NAFLD. Artificial intelligence (AI) and machine learning are becoming promising tools for detecting biomarkers from these datasets, extracting knowledge, interpreting systems biology, and helping with developing precision medicine. In this review, we summarize current evidence that supports the role of the gut–brain–immune axis in neurodegenerative and metabolic diseases, highlighting results gained with the utilization of multi-omics approaches. We will describe the key microbial, immune, and metabolic pathways involved in pathogenesis and therapeutic approaches including psychobiotics, tailored nutrition, modulation of the microbiome, and metabolite interventions, discussing future perspectives of the translation of the gut–brain–immune axis knowledge into clinical practice.

## 1. Introduction

The human gut harbors trillions of microorganisms that influence not only gastrointestinal physiology but also neurological, metabolic, and immune functions. Communication between the gut and the brain occurs through multiple interconnected pathways, including neural signaling via the vagus nerve and enteric nervous system, endocrine signaling through hormones such as cortisol and leptin, and immune-mediated signaling involving cytokines and inflammatory mediators. These bidirectional interactions collectively form the gut–brain axis and play important roles in regulating cognition, behavior, metabolism, and systemic homeostasis [1]. In addition, gut microbes produce several bioactive metabolites, including short-chain fatty acids (SCFAs), bile acids, and neurotransmitter precursors, which contribute to intestinal barrier integrity, immune regulation, and neurophysiological signaling [2]. The vagus nerve serves as a major bidirectional communication pathway, transmitting microbial and inflammatory signals from the gut to the central nervous system and regulating intestinal motility, immune responses, and neuroendocrine signaling.

The immune system is an essential component of the gut–brain axis, giving rise to the broader concept of the gut–brain–immune axis. Alterations in gut microbial composition (dysbiosis) can disrupt intestinal barrier function, increase gut permeability, and promote endotoxemia and systemic inflammation. These inflammatory changes may activate immune signaling pathways and contribute to chronic neuroinflammation and metabolic dysfunction [3]. Increased intestinal permeability permits translocation of lipopolysaccharides and inflammatory cytokines into systemic circulation, which may subsequently activate microglia and promote chronic neuroinflammation. Increased production of inflammatory cytokines, oxidative stress, and altered immunecell responses are increasingly recognized as important mediators linking gut dysbiosis with chronic disease progression. SCFAs such as butyrate, acetate, and propionate help maintain intestinal epithelial tight junctions, regulate microglial maturation, and suppress excessive inflammatory signaling. Dysbiosis-associated reduction in SCFA production may therefore compromise gut barrier integrity and promote systemic inflammation.

Emerging evidence strongly implicates gut–brain–immune dysregulation in neurodegenerative disorders such as Alzheimer’s disease (AD) and Parkinson’s disease (PD). Experimental studies in PD models treated with alpha-synuclein oligomers have demonstrated inflammation in both gastrointestinal and brain tissues, suggesting early involvement of the gut immune system in disease pathogenesis [3]. Gastrointestinal abnormalities and alpha-synuclein accumulation may occur nearly 10–15 years before the onset of motor symptoms, supporting the hypothesis of a gut-origin component in PD progression [4]. Similarly, alterations in gut microbial diversity have been associated with amyloid plaque deposition and cognitive decline in AD models, potentially mediated through inflammatory signaling pathways [5]. Increased intestinal permeability permits translocation of lipopolysaccharides and inflammatory cytokines into the systemic circulation, which may subsequently activate microglia and promote chronic neuroinflammation.

Similar mechanisms are also observed in metabolic disorders such as obesity and type 2 diabetes mellitus (T2DM), both of which are characterized by chronic low-grade inflammation. Dysbiosis-associated endotoxemia and inflammatory activation can impair pancreatic β-cell function, alter lipid metabolism, and promote insulin resistance and obesity. Importantly, metabolic and neurodegenerative disorders share several overlapping pathological mechanisms, including chronic inflammation, oxidative stress, mitochondrial dysfunction, inflammasome activation, and altered immune signaling pathways [6]. Chronic adipose tissue inflammation and metabolic endotoxemia may further promote systemic cytokine release, oxidative stress, and impaired insulin signaling, thereby contributing to neuroinflammatory and neurodegenerative processes [7]. These shared features suggest significant mechanistic overlap within the gut–brain–immune network and highlight the possibility of common therapeutic targets [8,9].

Recent advances in high-throughput multi-omics technologies have substantially improved our understanding of host–microbiome interactions. Approaches such as metagenomics, transcriptomics, metabolomics, proteomics, and single-cell sequencing allow for the comprehensive characterization of microbial communities, immune responses, metabolic pathways, and cellular heterogeneity at unprecedented resolution [10]. The integration of these datasets has accelerated biomarker discovery and systems-level understanding of disease mechanisms. Compared to single-omics approaches, integrated multi-omics analyses enable simultaneous evaluation of microbial composition, host gene expression, metabolic alterations, and immune signaling pathways, thereby providing a more comprehensive understanding of disease mechanisms. Emerging artificial intelligence (AI)-based analytical frameworks are increasingly supporting multi-omics data integration and precision medicine approaches [10,11].

Future directions are to develop robust, population-specific analyses. For example, the human populations in Europe, US, Africa, or Asia may have different gut microbiomes due to differences in lifestyle and diet. Longitudinal studies with different demographics and a deeper understanding of individual responses are essential. This review provides an overview of how the gut–brain–immune axis is connected with AD and PD and other neurodegenerative diseases and obesity and T2DM. It explains how multi-omics techniques are being utilized to gain greater insight into the interaction between these components and reviews current and future therapeutic and translational goals.

Rather than discussing these disorders as isolated entities, this review is organized around the shared mechanistic framework of the gut–brain–immune axis. Particular emphasis is placed on convergent pathways including chronic inflammation, microbial metabolite dysregulation, mitochondrial dysfunction, oxidative stress, and barrier impairment, while highlighting how multi-omics technologies and AI-driven integration approaches are advancing translational understanding across both neurodegenerative and metabolic diseases. AI and machine learning approaches can identify complex nonlinear relationships within large multi-omics datasets, thereby supporting biomarker discovery, predictive disease modeling, patient stratification, and precision medicine strategies.

Several previous reviews have independently examined gut microbiota dysbiosis, neuroinflammation, or microbiome-associated metabolic disorders. However, most have focused either on single diseases, isolated omics platforms, or conventional microbiome-centered mechanisms. The present review provides an integrated overview of the gut–brain–immune axis by combining evidence from neurodegenerative and metabolic diseases with emerging multi-omics technologies, including metagenomics, transcriptomics, metabolomics, proteomics, single-cell sequencing, and AI-assisted data integration. Particular emphasis is placed on shared mechanistic pathways and the translational potential of systems-level approaches for biomarker discovery and precision medicine. For this narrative review, literature searches were conducted using PubMed, Scopus, and Web of Science databases, focusing primarily on studies published between 2015 and 2025. Search terms included combinations of ‘gut–brain axis’, ‘gut microbiota’, ‘multi-omics’, ‘metagenomics’, ‘transcriptomics’, ‘metabolomics’, ‘proteomics’, ‘neurodegenerative diseases’, ‘metabolic diseases’, ‘microbiome’, ‘AI’, and ‘precision medicine’. Relevant original articles, experimental studies, clinical investigations, and recent review articles related to neurodegenerative and metabolic disorders were considered.

## 2. Overview of the Gut–Brain–Immune Axis

**Figure 1:**
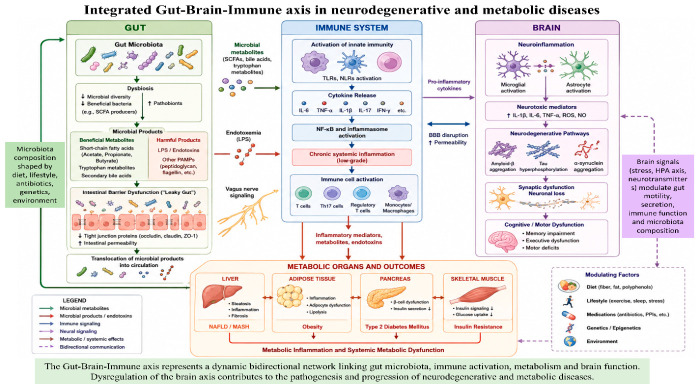
Integrated gut–brain–immune axis in neurodegenerative and metabolic diseases. The figure illustrates the bidirectional communication network linking gut microbiota, the immune system, metabolic organs, and the central nervous system (CNS). Gut microbial composition is influenced by diet, lifestyle, medications, genetics, and environmental factors. Beneficial microbial metabolites, including short-chain fatty acids (SCFAs), bile acids, tryptophan metabolites, and neurotransmitter precursors, contribute to intestinal barrier integrity, immune homeostasis, and neurophysiological signaling. In contrast, dysbiosis promotes intestinal barrier dysfunction (“leaky gut”), reduced tight junction protein expression, and translocation of microbial products such as lipopolysaccharides (LPS) into systemic circulation, leading to endotoxemia and chronic low-grade inflammation. The immune compartment highlights activation of innate and adaptive immune responses, including Toll-like receptor (TLR) and inflammasome signaling, cytokine release (IL-1β, IL-6, TNF-α, IFN-γ), and immunecell activation. These inflammatory mediators contribute to blood–brain barrier (BBB) disruption, oxidative stress, mitochondrial dysfunction, and neuroinflammation characterized by microglial and astrocyte activation. Neurodegenerative processes including amyloid-β aggregation, tau hyperphosphorylation, α-synuclein accumulation, synaptic dysfunction, neuronal loss, and cognitive or motor impairment are illustrated. The figure also demonstrates interactions with metabolic organs, including adipose tissue, liver, pancreas, and skeletal muscle, linking gut dysbiosis with obesity, insulin resistance, type 2 diabetes mellitus (T2DM), and NAFLD/MASLD. Neural and endocrine pathways, including vagus nerve signaling and stress-related hypothalamic–pituitary–adrenal (HPA) axis modulation, further integrate gut–brain–immune communication. Overall, the figure summarizes how dysregulation of interconnected microbial, immune, neural, and metabolic pathways contributes to the pathogenesis of neurodegenerative and metabolic diseases.

The gut–brain–immune axis comprises the interconnected gut microbiota, gut, CNS, and immune system, which exchange biochemical, neural, and immunological signals that maintain homeostasis rather than operate independently as an integral system. Disruption of this dynamic axis promotes chronic inflammation, neurodegenerative, and metabolic disorders. The gut–brain–immune axis represents the interconnected communication network between the gut microbiota, gut, CNS, and the immune system (Figure 1). This bidirectional communication network dynamically integrates neural pathways such as the vagus nerve and enteric nervous system, endocrine signaling through hormones and the hypothalamic–pituitary–adrenal axis, immune-mediated cytokine signaling, and microbial metabolites including SCFAs and bile acids.

### 2.1. Gut Microbiota and Host Homeostasis

The gut microbiota in the gastrointestinal tract of humans contains trillions of bacteria, archaea, fungi, and viruses, which play numerous roles like digestion, vitamin production, training of the immune system, and the maintenance of gut epithelial integrity [12]. In addition to these benefits, gut bacteria also create a variety of biologically active metabolites that have implications for remote organs of the host, including the brain.

A variety of microbial products have received significant study, including SCFAs such as butyrate, acetate, and propionate, all made through the bacterial fermentation of fiber; they contribute to the maintenance of gut epithelial integrity, regulation of inflammatory responses in the gut, and proper microglial development in the brain [13]. Gut microbes and their metabolites also interact closely with intestinal epithelial cells and immune cells to maintain immune tolerance and mucosal homeostasis. Butyrate in particular has anti-inflammatory and neuroprotective effects by serving as an inhibitor of histone deacetases and regulators of immune signaling pathways, but gut microbes play many more roles including the metabolism of bile acids [14]. Bile acid signaling through receptors such as FXR and TGR5 further contributes to immune and metabolic regulation. Catabolism of the amino acid tryptophan leads to the creation of the neurotransmitter acetylcholine. Microbial regulation of tryptophan metabolism and the kynurenine pathway may influence serotonin synthesis, neuroinflammation, and immune signaling [15]. Under normal circumstances, the gut barrier functions to prevent migration of these bacteria or products into the blood. However, changes to the normal microbial composition or dysbiosis result in the failure of intestinal epithelial tight junctions, making the gut leaky, thereby increasing migration of bacterial products such as lipopolysaccharides (LPS), inflammatory cytokines, and immune cells into the bloodstream and thereby stimulating chronic inflammation and activating the immune system. These changes are well documented in metabolic and neurologically focused dysbiosis models [8].

### 2.2. Neural Communication Between the Gut and Brain

Gut–brain crosstalk is a complex bidirectional dialogue, carried out via multiple mechanisms involving the vagus nerve, the enteric nervous system, the spinal pathways, and the neuroendocrine system. Enteric glial cells also contribute to neuroimmune signaling by regulating intestinal barrier integrity and inflammatory responses. The vagus nerve, among the main pathways in gut–brain signaling, conveys sensory signals from the gastrointestinal system toward the brainstem and governs activities of the gut such as transit, and also controls the inflammatory response [11]. Microbiota-produced molecules impact neuronal activity either directly or indirectly. Many species create some neurotransmitters or neuroactive molecules such as GABA, serotonin, dopamine, and acetylcholine. These microbial metabolites may further influence synaptic plasticity, neuronal signaling, and neurobehavioral regulation [16]. Most peripheral neurotransmitters are unable to cross the blood–brain barrier properly, but they do modulate neural activity via either parasympathetic nerve activation or the immune system. Almost 90% of the serotonin in our body is synthesized in the intestine, highlighting the functional relationship between the physiology of intestine and regulation of neurobehavior.

The hypothalamic–pituitary–adrenal axis forms another key component of the gut–brain–immune system. Psychological stress is a trigger that increases HPA axis activity and induces glucocorticoid release and changes in gut permeability, as well as shifts in microbiota composition. Furthermore, changes in gut flora are linked to increased vulnerability of an animal’s stress response and endocrine responses, thereby proving again that the two sides of the system influence one another [17].

### 2.3. Immune Crosstalk and Neuroinflammation

The immune system is one of the main means of mediating between the microbial world and the brain. The intestinal mucosal barrier contains a large population of innate and adaptive immune cells that continuously interact with gut microbiota and microbial metabolites to maintain immune homeostasis [18]. In a healthy state, this keeps the immune system and gut lining working under homeostasis, with controlled and healthy levels of inflammation; a dysbiotic state of the gut, however, could mean an inability to produce enough of certain molecules and produce enough inflammatory molecules (and in some cases, enough pro-inflammatory ones), which may travel into the systemic circulation and then into the brain. Chronic systemic inflammation is known to drive microglial activation and cause neuronal degeneration, insulin resistance, and metabolic disease [19]. Microglia (resident brain immune cells) responds directly to inflammatory changes in the blood; these are known to damage neurons with sustained inflammation. Astrocyte activation, peripheral immune cell infiltration, and cytokine-mediated pathways involving IL-6, TNF-α, and IL-1β further contribute to chronic neuroinflammation and inflammasome-associated neurotoxicity [20].

Studies have found that microbial metabolites may have both anti- and pro-inflammatory effects. Some, like SCFAs, generally act in an anti-inflammatory manner, whereas endotoxins such as LPS trigger Toll-like receptors and nuclear factor-kappa B (NF-kB), triggering pro-inflammatory signaling and increasing both reactive oxygen species and mitochondrial damage seen in neurodegenerative diseases such as Huntington’s and Parkinson’s and metabolic disorders like obesity and diabetes.

### 2.4. Blood–Brain Barrier Dysfunction and Systemic Effects

The blood–brain barrier (BBB) acts as a protective mechanism for the CNS from blood-borne toxins and inflammatory substances. Emerging research points to the involvement of gut dysbiosis and systemic inflammation in weakening the BBB’s function. Disruption of tight junction proteins, endothelial dysfunction, oxidative stress, and circulating endotoxins such as lipopolysaccharides may further increase BBB permeability and facilitate neuroinflammatory damage [21]. This impairment allows for more substantial passage of inflammatory cytokines and bacterial components to the brain tissue, potentially contributing to neuroinflammation [22]. Increased BBB permeability has been found to occur in diverse diseases, such as Alzheimer’s and MS. Impaired integrity of the BBB is associated with continued neuroinflammation through the activation of microglia cells, resulting in further astrocytosis and creating an inflammatory cascade. The gut–brain axis interacts closely with inflammatory pathways that occur in non-neurologic and non-gastrointestinal tissues such as the liver, pancreas, and adipose tissues. The gut–brain axis is considered an important regulating axis underlying chronic disease development instead of being specific to the gut or brain.

## 3. Integrative Multi-Omics and AI Approaches in Gut–Brain–Immune Axis Research

Given the complex and interconnected nature of the gut–brain–immune axis, advanced analytical approaches are required to comprehensively evaluate microbial composition, immune responses, metabolic signaling, and cellular heterogeneity. Recent advances in high-throughput multi-omics technologies have enabled systems-level characterization of host–microbiome interactions and substantially improved understanding of neurodegenerative and metabolic disease mechanisms. With recent progress in high-throughput omics technologies, such an analytical shift toward systems-level characterization of host–microbiome interactions at multi-level layers in neurodegenerative diseases or metabolic syndrome has opened up and, more recently, tremendously advanced the field.

### 3.1. Metagenomics and Microbial Community Profiling

Metagenomics is a fundamental approach in microbiome science. DNA extracted from complex microbiome samples, along with extensive knowledge of genome sequences in public databases, through next-generation sequencing generates a massive amount of DNA sequencing data, enabling comprehensive profiling of microbial diversity (taxa) and functional genes within a given community. Methods such as 16S ribosomal RNA sequencing, which identifies bacterial taxa by sequencing universally, conserved 16S rRNA genes, and whole-genome shotgun sequencing (WGSS), which sequences DNA from the entire community, are frequently employed for characterizing the gut microbial diversity and composition. Despite their utility, these approaches have important limitations, including limited species-level resolution in 16S rRNA sequencing, inability to differentiate viable from non-viable microorganisms, batch effects, and difficulties in standardizing microbiome datasets across studies and sequencing platforms [23]. Metagenomic studies have identified distinct microbial profiles in numerous neurological and metabolic disorders, such as the reduced presence of butyrate-producing *Faecalibacterium*, *Roseburia,* and *Prevotella* species in obesity, T2DM, and neurodegenerative disorders such as Parkinson’s and Alzheimer’s diseases [24], alongside increased abundance of pro-inflammatory taxa such as *Bacteroides*, which have been associated with increased systemic endotoxemia and inflammatory activation. Not only will metagenomics identify bacteria within gut microbiomes but it may also predict genes and functional metabolic pathways—such as amino acid and bile acid metabolism and biosynthesis of small-molecule drugs, neurotransmitters, vitamins, and inflammatory mediators—that carry out these functions in these specific gut microbes. Gut microbiome analysis may prove beneficial for gut–brain and gut–immune communication research because gut microbial composition alone does not correlate to the biological functions that play roles in gut diseases [25].

### 3.2. Transcriptomics and Host Immune Responses

Host transcriptomics primarily evaluates gene-expression changes in host tissues and immune cells, whereas metatranscriptomics analyzes actively expressed microbial genes within the microbiome. In contrast, single-cell transcriptomics enables characterization of gene-expression patterns at the individual-cell level, allowing identification of cellular heterogeneity and cell-specific responses [26,27]. The study of transcriptomics (bulk RNA sequencing and microarrays) tells us about the genes in host tissue that are activated or inactivated during microbiota disorders and inflammation. Transcriptomic technologies have been utilized in many studies to highlight immune activation, inflammatory signaling in the brain, and metabolic alterations both in peripheral locations (for example, the gut) and the CNS. Multiple studies have shown upregulation of inflammatory genes that promote nuclear factor-kappa B (NF-κB), inflammasome activation, cytokine production, and the activity of oxidative stress enzymes in patients with chronic conditions, such as metabolic and neurodegenerative disorders [11]. Additional pathways including JAK/STAT signaling, interferon-mediated responses and mitochondrial stress response pathways have also been implicated in neuroimmune and metabolic dysregulation [28]. Furthermore, transcriptomic profiling has identified pathways linked to the activation of resident microglia and the function of mitochondria and insulin signaling that are out of balance. By merging transcriptomics and metagenomics results, new insights into possible host–microbe crosstalk during pathogenesis become apparent. Gut microbiome dysbiosis and changes in immune cell expression are, for instance, found to be in sync with altered expression of intestinal barrier integrity, inflammatory cell trafficking, and neuroimmune response signaling pathways. These combined data offer further insights into the underlying biological mechanisms, far beyond taxonomic links with specific microbes.

### 3.3. Metabolomics and Microbial-Derived Signaling Molecules

Metabolomics investigates the identification of tiny molecules and metabolic intermediates found in samples. The gut microbiota’s metabolites are among the primary mediators of gut–brain communication, so metabolomics is frequently used in the field. Short-chain fatty acids, secondary bile acids, indole compounds, trimethylamine N-oxide (TMAO), and tryptophan metabolites, among others, originate from gut microbes and have effects on inflammation, neuronal communication, and metabolic control [29]. Emerging lipidomics approaches, including studies of endocannabinoid signaling and oxidative stress-related lipid metabolites, are also providing important insights into neurodegeneration, inflammation, and metabolic dysfunction. Patients with Alzheimer’s disease, Parkinson’s disease, obesity, and type 2 diabetes (T2DM) all exhibit altered metabolite levels, implying that there might be shared metabolic disruptions that connect these illnesses [30].

Tryptophan metabolism is fascinating since it influences bacterial growth and its regulation in neurotransmitter and hormone systems, namely, via serotonin production, serotonin degradation, and tryptophan kynurenine metabolism (kynurenine pathway). Studies have shown that changes in tryptophan metabolites are correlated with the onset and manifestation of depression, cognitive function, and metabolic inflammation. Likewise, reduced levels of butyrate and other short-chain fatty acids (SCFAs) are linked to reduced integrity of the gut barrier and the amplification of inflammatory processes in other systems. In relation to microbiome profiling methods like sequencing (taxonomic and functional), metabolomics is more representative of direct microbial metabolism of food and can track the functional contribution of microbes and metabolites of gut microbes to neuroinflammation and other pathological effects observed at distant sites, i.e., throughout the gut–brain axis [31].

### 3.4. Proteomics and Inflammatory Signaling Networks

Proteomics allows for the comprehensive study of the proteins, cytokine networks, enzyme activities, and signaling pathways that are keys to pathogenesis. These proteomic analyses have been performed using diverse sample sources, including plasma, cerebrospinal fluid (CSF), brain tissue, intestinal tissue, and experimental animal models, each providing distinct biological and clinical insights. Since proteins are the effector molecules of gene expression, proteomics offers indispensable perspectives on dynamic cell signaling as well as the regulation of immunity. Studies of proteomic signatures in neurodegenerative disease show the increased involvement of immune modulatory molecules including numerous mediators of inflammation (e.g., IL-6, TNF-α), the complement system, and synapse-related proteins (e.g., SNAP-25), as well as evidence of heightened oxidative stress [32]. Proteomics in the field of metabolic disorders is frequently characterized by the dysregulation of insulin signaling proteins, key inflammatory molecules (e.g., IL-1β, IL-8), and mitochondrial enzymes that manage energy production (e.g., ATP synthase subunit β) [33]. Integrating proteomics with metabolomics has allowed for further assessment of how microbial metabolites modulate immune signaling and directly influence neurons. Such comprehensive approaches may ultimately enable the identification of potential circulating protein markers that serve roles as diagnosticians or prognostics of various conditions.

### 3.5. Single-Cell and Spatial Omics Technologies

Conventional bulk omics approaches average gene expression across cell mixtures and, hence, it is challenging to resolve cell-specific responses associated with disease pathogenesis. Single-cell sequencing techniques allow for profiling the transcriptome of individual cells within a population, enabling the identification and annotation of distinct immune cells and neurons that participate in disease pathways. Single-cell RNA sequencing analysis has unveiled the significant diversity of microglia, astrocytes, and intestinal epithelial cells and also immune cell populations present in chronic neuroinflammation, as well as those associated with metabolic disorders [34], helping improve understanding of how cells interact in the gut–brain–immune axis and which cells contribute to inflammatory processes of particular diseases. Spatial transcriptomics provides a parallel approach by integrating genomic analysis with spatial information and tissue morphology, which allows for the visualization of inflammatory sites and microbe–host interactions, as well as neuroimmune responses within tissues. Although still developing, the use of spatial omics in the study of disease-related tissues promises to further increase the understanding of niche-specific features and processes contributing to pathologies [35]. However, these technologies remain limited by their high cost, computational complexity, tissue preservation requirements, and challenges related to spatial resolution and cross-platform data integration. Combining spatial transcriptomics with advanced imaging approaches may further improve characterization of tissue-specific neuroimmune interactions.

### 3.6. Artificial Intelligence and Multi-Omics Integration

As large amounts of multi-omics data are produced, sophisticated analytical tools are required for the interpretation of this complex data. Several recent works have applied AI or machine learning approaches to integrate metagenomic, transcriptomic, metabolomic, and clinical datasets for identifying potential biomarkers and improving applications in personalized medicine [36,37]. Artificial intelligence and machine learning algorithms are increasingly being used to integrate metagenomic, transcriptomic, metabolomic, and clinical data in identifying new biomarkers for disease prediction and finding personalized therapies for different patient populations. These artificial intelligence-based analyses allow researchers to discover nonlinear complex patterns or interactions in the data that cannot be addressed through standard statistical methods. However, important limitations remain, including risks of model overfitting; limited availability of external validation cohorts; challenges in interpreting AI-derived biomarkers; and ethical concerns related to patient privacy, data integration, and clinical implementation. Several examples of machine learning applications in health have been published: the identification of diagnostic and predictive markers to detect disease risk and progression in metabolic diseases or to identify specific microbiome-associated biomarkers and stratification strategies of patient groups in Alzheimer’s disease and other neurological conditions [38]. The primary challenges facing the multi-omics era are the standardization and reproducibility of methodologies, as well as the integration of heterogeneous data acquired from various populations and platforms. Despite these obstacles, AI-assisted multi-omics will be crucial in future precision medicine strategies targeting the gut–brain axis (Table 1).

## 4. Gut–Brain–Immune Axis in Neurological and Neuropsychiatric Disorders

Neurodegenerative diseases are increasingly associated with gut microbiota dysbiosis, chronic neuroinflammation, immune dysregulation, mitochondrial dysfunction, and altered microbial metabolite signaling. Multi-omics studies have identified overlapping inflammatory and metabolic pathways linking gut–brain–immune dysfunction with disorders such as Alzheimer’s disease and Parkinson’s disease.

### 4.1. Alzheimer’s Disease

It is no mystery that Alzheimer’s is the most common neurodegenerative illness worldwide and is identified by a gradually worsening inability to think properly, large extracellular amyloid plaque clumps, hyperphosphorylated tau protein, impaired communication between neurons, and ongoing neuroinflammation. Moreover, recent research has provided considerable evidence that changes in gut microbiota alterations are implicated in AD pathogenesis. Different groups have found lower microbial diversity in AD patients along with decreased amounts of certain bacteria known for reducing inflammation [39]. Fewer gut bacteria produce short-chain fatty acids. This might hurt the health of the intestinal lining and result in systemic inflammation. Greater gut permeability allows endotoxins from gut microbes—like LPS—to migrate into the circulation. This may promote systemic immune activation in the rest of the body, which further activates neuroinflammation in the brain. Chronic inflammatory signaling may promote microglial priming and NLRP3 inflammasome activation, thereby amplifying neurotoxicity and amyloid-associated pathology. Neuroinflammation is a major contributor to the ongoing deterioration in AD. Peripheral cytokines like IL-1β, IL-6, and TNF-α can travel across or compromise the BBB [40]. This in turn stimulates microglia cells (the brain’s resident immune cells) and glial cells (the brain’s supporting cells) to become more active inside the central nervous system. If this stays ongoing, those immune cells cause more oxidative stress and damage neurons. Chronic peripheral inflammation may also promote tau hyperphosphorylation, impair synaptic plasticity, and contribute to progressive neuronal dysfunction. Moreover, chronic activation is associated with additional amyloid plaques being created [41]. By examining different chemicals found in the bodies of AD patients, we have also noticed changes in bile acids, tryptophan byproducts and microbial-produced lipids. Some chemicals created by gut microbes might have a direct impact on amyloid creation and the systems by which neurons interact with one another. Microbial amyloids may also contribute to amyloid cross-seeding mechanisms and further stimulate inflammatory responses [42]. The combination of findings from these various studies has demonstrated that gut bacterial imbalances and problems with the immune system might be linked together in Alzheimer’s development, rather than simply being results of the disease.

### 4.2. Parkinson’s Disease

Parkinson’s disease (PD) primarily refers to dopaminergic neuronal loss within the substantia nigra. Parkinson’s disease is increasingly recognized as a multisystem disorder involving both central and enteric nervous system pathology [43]. Some signs of Parkinson’s disease can predate the emergence of motor symptoms by many years, suggesting a partial origin of Parkinson’s disease from the gut. One possible model states that pathological aggregates of misfolded alpha-synuclein originate in the enteric nervous system before spreading to the brain through the vagus nerve [4]. This concept is consistent with Braak’s hypothesis, which proposes that pathological α-synuclein aggregation may originate in the enteric nervous system and propagate to the CNS through vagal pathways. Gastrointestinal dysfunction, particularly constipation, is also recognized as an important prodromal feature of PD [44]. Evidence that aligns with this idea is the observation of abnormal gut bacteria compositions as well as increased evidence of intestinal inflammation among PD patients. Metagenomic research often shows a decline in the proportion of bacteria beneficial for SCFAs and, conversely, increased proportions of harmful bacteria that induce pro-inflammation. These reduced SCFA proportions can potentially undermine the structural integrity of the intestinal barrier and affect the development of microglia, which could trigger neuroinflammation. Simultaneously, greater absorption of lipopolysaccharide into the bloodstream along with the release of cytokines involved in inflammation leads to the NF-κB pathway becoming activated, along with the occurrence of oxidation stress. Other findings from transcriptomics and proteomics have implicated disordered immune response pathways involving mitochondria and immune signaling, as well as inefficient cellular debris management in PD [45]. Impaired mitochondrial quality-control pathways and autophagy dysfunction may further contribute to α-synuclein accumulation and neuronal injury. In addition, gut microbial metabolites may influence dopamine metabolism and neurotoxicity, further linking intestinal dysbiosis with PD progression [46]. In addition, gut microbe metabolites could alter dopamine metabolism and neurochemical messaging, providing yet another pathway linking intestinal microbial disturbances to brain degeneration. Some studies incorporating microbiome, metabolomics, and host gene expression have revealed markers associated with Parkinson’s disease that can be used for diagnostic purposes. Causality is not entirely understood, but substantial research does strongly support the link between gut–brain–immune processes in Parkinson’s disease progression [47].

### 4.3. Multiple Sclerosis

MS is a chronic inflammatory autoimmune disease with inflammation of myelin in the CNS. Experimental autoimmune encephalomyelitis (EAE) animal models have further demonstrated that alterations in gut microbiota composition can influence neuroinflammation and disease severity in MS [48]. It differs from Alzheimer’s disease (AD) and Parkinson’s disease (PD) in its primarily immune-mediated nature and importance for the gut–brain axis. We observed specific gut microbial signatures of dysbiosis among MS patients in that there were increases in pro-inflammatory microorganisms capable of driving the differentiation of Th17 cells that produce inflammatory cytokines and marked decreases in anti-inflammatory bacteria generally considered beneficial for the regulation of CNS immune responses [49]. Microbial factors may affect tight junction protein expression to impact the permeability of the blood–brain barrier, driving microglial activation and inducing the differentiation of inflammatory Th17 cells and decreasing regulatory T cell activity that reduces neuroinflammation. In contrast, dysbiosis may lead to an increase in lipopolysaccharide from Gram-negative bacteria, which can activate microglial cells and promote Th1 cell activation, leading to inflammation. RNA sequencing and single-cell sequencing technologies reveal an abundance of heterogeneity in MS. Molecular mimicry between microbial antigens and host proteins may further contribute to autoimmune activation in MS. Increased BBB permeability and trafficking of gut-primed immune cells into the CNS may additionally promote neuroinflammatory and demyelinating processes [50]. Activated microglia, astrocytes, macrophages, and activated autoreactive T lymphocytes all contribute to inflammation, the ultimate destruction of myelin, and subsequent injury to the CNS. As such, a combination of multi-omics approaches to combine microbiome and immune profiles has become of greater significance to investigate disease heterogeneity and pinpoint new therapeutic targets.

### 4.4. Depression and Neuropsychiatric Disorders

While not traditionally viewed as “neurodegenerative diseases”, depression and most neuropsychiatric disorders also show a close relationship to the gut microflora and its dysbiosis, chronic system inflammatory activation, and an array of different forms of neuroinflammatory reaction. Metabolic dysregulation of neurotransmitters and hypothalamic–pituitary–adrenal axis (HPA) axis dysregulation are most frequently associated with depressive disorders. The gut microbiota influences neurotransmitters (i.e., serotonin, dopamine), GABA or tryptophan metabolism, and the production of metabolites derived from tryptophan, as these contribute to mood [51]. It also may activate tryptophan to its neurotoxic metabolite forms associated with depression via activation of the kynurenine pathway in chronic inflammation originating from dysbiosis flora [51]. Metatranscriptomic, metabolomic, and metagenomic sequencing in cohorts in different locations has globally confirmed that depression has associated microbial signatures that include some microbial groups with a reduced abundance but not to the degree that it suggests significant loss, as well as an increased presence of other specific microbes. A variety of metabolic compounds can also be produced, which are linked to depression, but findings are not exactly consistent among different depression study populations. These inconsistencies may partly reflect differences in diet, medication exposure, geography, lifestyle factors, study design, and population heterogeneity, which continue to complicate the interpretation of microbiome findings in psychiatric disorders. However, the evidence is overwhelming that neuroinflammation and microbiome-related metabolic disorders may contribute to the pathology of psychiatry. Degenerative and neuropsychiatric diseases share important mechanistic components including microflora, inflammation, mitochondrial dysfunction, and chronic inflammatory signaling as part of common pathobiology and further application of multi-omics techniques will help better understand this intricate biological communication, leading to novel therapeutic treatments (Table 2).

## 5. Gut–Brain–Immune Axis in Metabolic Diseases

Metabolic diseases like obesity, T2DM, and NAFLD are a group of chronic inflammatory disorders with strong involvement of the gut microbiome as well as immune cells, both having crucial functions, roles, and mechanisms in these metabolic disorders. Metabolic diseases such as obesity, type 2 diabetes mellitus (T2DM), and NAFLD are increasingly associated with gut microbiota dysbiosis, immune dysregulation, and chronic low-grade inflammation. Alterations in intestinal barrier integrity, microbial metabolites, and endotoxemia may contribute to impaired metabolic signaling, insulin resistance, and systemic inflammatory activation within the gut–brain–immune axis [52].

### 5.1. Obesity

The presence of excess fat accumulation defines obesity; nevertheless, the inflammation inherent in metabolic dysfunction serves as another crucial hallmark. Certain microbial compositions have become linked to the prevalence of obesity, although these patterns may differ among demographics and diets. Some studies have reported reduced microbial diversity and alterations in the Firmicutes/Bacteroidetes ratio in obesity, although findings remain inconsistent across different populations, diets, and study designs [53]. Food can disrupt balance, and this dysbiosis may cause increased absorption by the intestines, enabling more microbial endotoxins such as lipopolysaccharides to travel into the blood, a process called metabolic endotoxemia, thereby activating cell-surface-mounted Toll-like receptor pathways to induce the release of inflammatory mediators such as tumor necrosis factor-alpha (TNF-α), IL-1β, and IL-6 [54]. Persistent, but minimal, inflammation leads to adipose tissue dysfunction, resistance to insulin, and changes in the body’s processing of fats. Adipose tissue macrophage polarization toward a pro-inflammatory phenotype, leptin resistance, and hypothalamic inflammation may further disrupt appetite regulation, energy homeostasis, and neuroimmune signaling in obesity [55].

There are additional microbes affecting the regulation of hunger and energy use. SCFAs affect the secretion of hunger-inducing gut hormones, such as glucagon-like peptide-1 and peptide YY, influencing appetite and glucose metabolism. Additionally, gut microbial action on bile acid metabolism influences how lipids in the body are regulated, and include inflammatory responses through Farnesoid X receptor and Takeda G protein-coupled receptors in the cell. Activation of FXR and TGR5 signaling pathways may influence glucose homeostasis, lipid metabolism, inflammatory responses, and neuroimmune communication within the gut–brain axis. Transcriptomic and metabolomic data reveal several identical inflammatory pathways that occur in obesity and neurodegenerative disorders; common examples are the activation of the transcription factor NF-κB, oxidative stress, and failure of mitochondria. Research findings indicate that the inflammation connected to obesity has the ability not just to increase metabolic disorder symptoms but also to lower cognition and spark brain inflammation as well.

### 5.2. Type 2 Diabetes Mellitus

Accumulating evidence suggests that gut microbiota dysbiosis contributes to metabolic dysfunction in T2DM. With increased frequency, metagenomic studies performed in T2DM patients with dysbiotic gut microbiomes have reported fewer helpful butyrate-producing bacteria, as well as a higher abundance of some inflammation-associated opportunistic bacteria [56]. There seems to be evidence that fewer short-chain fatty acids are produced, leading to weaker gut barrier functions and an even further escalation of inflammatory processes that contribute to insulin resistance.

T2DM patients also generally suffer from much higher levels of ongoing chronic immune activation. These increased inflammatory cytokines result in impaired insulin signaling and therefore poor handling of sugars within the liver, muscle, and adipose tissues. Gut microbial metabolites may also influence incretin signaling pathways such as those involving glucagon-like peptide-1 (GLP-1), insulin sensitivity, and glucose homeostasis. In addition, mitochondrial dysfunction and oxidative stress within pancreatic β-cells may further contribute to impaired insulin secretion and metabolic dysregulation [57]. Inflammasomes and oxidative stress activation lead to pancreatic Beta cell loss and a worse metabolic profile. Bile acid metabolism is disturbed, BCAAs are increased, and Trp metabolism is altered. Elevated branched-chain amino acids (BCAAs) have been associated with impaired insulin signaling, mitochondrial dysfunction, and chronic metabolic inflammation, thereby contributing to insulin resistance and glucose dysregulation [58]. Some of these alterations lead to an improvement in peripheral insulin sensitivity, but others alter metabolism centrally. Emerging evidence also suggests a bidirectional relationship between gut microbiota alterations and metabolic dysregulation in T2DM. Multi-omics integration between microbiome, transcriptome, and metabolome data has pointed to a few possible markers for early diagnosis, and even some promising models using machine learning to define metabolic risk factors solely based on their microbiome features.

### 5.3. Non-Alcoholic Fatty Liver Disease

Metabolic dysfunction-associated steatotic liver disease (MASLD; historically termed NAFLD) is closely associated with obesity, insulin resistance, and gut microbial dysbiosis [59]. The gut–liver axis is now considered the most important anatomical structure responsible for transferring intestinal contents and their metabolic mediators to the liver. Intestinal dysbiosis promotes changes in intestinal barrier permeability that help the passage of circulating bacterial endotoxins and inflammatory components into the liver via portal circulation, leading to liver inflammation and fibrosis. Activation of Kupffer cells, oxidative stress, and chronic cytokine signaling further contribute to hepatic inflammation, fibrosis progression, and metabolic dysfunction. Moreover, increased permeability and a change in the balance of microbiota with dysbiosis induce profound changes in bile acids, choline breakdown, and SCFA production by bacterial fermentation, which influence lipid metabolism [59]. BBB dysfunction may additionally facilitate accumulation of inflammatory mediators and amyloid-related pathology within the CNS. Some of the inflammation and metabolic pathways that lead to MASH, such as enhanced mitochondrial function defects and increased mitochondrial and cytoplasmic lipid droplets as well as increased cytokine production, are similar to those in neurodegenerative diseases. Thus, MASH is currently seen as being on a wider scale in metabolic and inflammation-related illnesses involving multiple anatomical compartments via the gut–brain axis as well as the gut–immune interface [59].

### 5.4. Shared Mechanisms Linking Metabolic and Neurodegenerative Disorders

Chronic inflammation, mitochondrial failure, oxidative stress, inhibited cellular cleansing (autophagy), and modified production of bacterial metabolites seem to have been implicated across several metabolic and neurodegenerative disorders related to the gut–brain axis and immune interactions. Additional shared mechanisms include impaired autophagy, endoplasmic reticulum stress, mitochondrial quality-control dysfunction, and systemic immunometabolic dysregulation. The concept of ‘metaflammation’—chronic low-grade metabolic inflammation—has also emerged as an important link connecting metabolic and neurodegenerative disorders. Circadian rhythm disruption and sleep disturbances have also been increasingly associated with gut microbiota dysregulation, chronic inflammation, impaired metabolic homeostasis, and neurodegenerative processes [60]. Also, metabolic disorders may promote cognitive deficit as a result of lowered utilization of glucose in neural tissues and heightened local inflammation, whereas neuroinflammation can affect the metabolic state. These findings suggest potential mechanistic overlap between metabolic and neurodegenerative disorders from the perspective of the host brain. The same holds for recent analyses through multi-omics technologies such as genome-wide association (GWA) studies, which also highlight interconnected networks of genes involved in microbial ecology, immunity, and metabolism [61]. This may be an advantage for treatment aiming at common features shared across multiple chronic conditions (Table 3).

## 6. Shared Molecular Mechanisms Linking Neurodegenerative and Metabolic Diseases

Neurodegenerative and metabolic diseases are distinct in a medical context, although evidence mounts that they possess similar pathophysiological underpinnings. The gut–brain–immune nexus serves as an important integrative model to explain this convergence of mechanisms, as disruptions in the gut ecosystem (dysbiosis) can impair metabolism and trigger inflammation throughout the body and brain. Mechanisms that underpin these comorbidities include sustained inflammation, the loss of intestinal barrier integrity, modifications to microbial metabolites, mitochondria malfunction, the exacerbation of damage from oxidants, and disruptions of insulin action.

### 6.1. Chronic Low-Grade Inflammation

One commonality linking obesity, T2DM, Alzheimer’s, and Parkinson’s is a degree of chronic, low-grade inflammation. The inflammatory factors released include TNF-α, IL-1β, and IL-6. Such cytokine signaling hinders insulin receptor-dependent pathways in T2DM and is associated with damage to brain cells through microglial and astrocytic activation in neurodegenerative conditions [64]. Additional mechanisms including NLRP3 inflammasome activation, microglial priming, astrocyte-mediated neuroinflammation, and systemic immunometabolic signaling may further contribute to chronic inflammatory and neurodegenerative processes. The concept of metaflammation has also emerged as an important feature of obesity-associated chronic low-grade inflammation [65]. Gut dysbiosis often increases chronic inflammation by raising intestinal permeability, allowing for greater passage of gut microbiome metabolites like lipopolysaccharide and triggering Toll-like receptor and NF-κB signaling. This process exacerbates both metabolic endotoxemia and neuroinflammation, acting as a link between peripheral disease state and brain injury [62,66]. Persistent Toll-like receptor and NF-κB activation may impair insulin signaling, increase blood–brain barrier permeability, and promote chronic neuroinflammation, thereby contributing to both metabolic dysfunction and neurodegeneration [67].

### 6.2. Gut Barrier and Blood–Brain Barrier Dysfunction

The gut barrier and the BBB are major components of the immune system’s barriers, which are disrupted by gut–brain–immune dysregulation. Healthy barrier function of the gastrointestinal tract is key to the prevention of microorganisms and their toxins entering the bloodstream, while a properly functioning BBB is important for the protection of the brain from inflammatory molecules circulating in the blood and for preventing entry of such cells/molecules into CNS tissues [68]. Gut microbial dysbiosis, a poor Western diet, chronic stress, obesity, or inflammations are among the factors that can disrupt gut and BBB integrity. Disruption of tight junction proteins including occludin, claudins, and zonula occludens-1 (ZO-1), together with endothelial dysfunction and oxidative stress, may further impair barrier integrity. Microbial endotoxins such as lipopolysaccharides can additionally increase BBB permeability and promote neuroinflammatory injury [69]. Increased intestinal permeability is related to amplified inflammation, while the BBB is further compromised, allowing cytokines, lipopolysaccharides, and inflammatory cells to penetrate CNS tissues. This process increases the reactivity of microglia and induces synaptic dysfunction and neuronal damage. Barrier failure serves as a mechanism to both initially and further accelerate chronic neuroimmune disorders involving the cooccurrence of metabolic inflammation and neuroinflammation. However, whether BBB dysfunction represents an initiating factor in neuroinflammatory disease or develops secondary to systemic inflammation and metabolic dysregulation remains an area of ongoing investigation.

### 6.3. Microbial Metabolites as Signaling Mediators

Short-chain fatty acids such as butyrate, propionate, and acetate are probably the most important metabolites mediating communication between the gut microbiota and the brain and the immune system. For example, these fatty acids regulate immune tolerance and intestinal barrier function and influence lipid metabolism, glucose metabolism, and microglial development in the brain. Reduced concentrations of these beneficial SCFAs have also been observed in several neurodegenerative and metabolic diseases such as metabolic dysfunction-associated steatotic liver disease and metabolic dysfunction-associated steatohepatitis (MASLD/MASH), and it is plausible that inflammation arises because of an altered imbalance of microbiota and their beneficial metabolites [13]. In addition to SCFAs, metabolites of the amino acid tryptophan such as serotonin kynurenine metabolites and indoles are important mediators linking microbial metabolism and brain function, while bile acid metabolites affect a broad range of processes spanning lipid and glucose metabolism, inflammation, and signaling within neurons. The kynurenine pathway is particularly important, as dysregulated kynurenine metabolism may contribute to oxidative stress, neurotoxicity, immune dysregulation, and chronic neuroinflammatory processes [70]. Additional metabolites including branched-chain amino acids (BCAAs) and endocannabinoid-related metabolites have also been implicated in insulin resistance, metabolic inflammation, neuroimmune regulation, and gut–brain signaling. In contrast to the generally protective effects of SCFAs, certain microbial metabolites such as trimethylamine N-oxide (TMAO) and altered secondary bile acids have been associated with chronic inflammation, metabolic dysfunction, and neurodegenerative disease progression [70]. Thus, distinct profiles of altered gut metabolites can potentially link changes in intestinal health with consequences in both peripheral metabolic tissues and CNS tissues [63,71].

### 6.4. Mitochondrial Dysfunction and Oxidative Stress

The common traits of both metabolic and neurodegenerative diseases are mitochondrial dysfunction and oxidative stress. Additional mechanisms including impaired mitochondrial biogenesis, defective mitophagy, endoplasmic reticulum stress, oxidative phosphorylation dysfunction, and mitochondrial DNA damage may further contribute to chronic inflammation, neuronal injury, and metabolic dysregulation [72]. Mitochondrial stress leads to insulin resistance and poor energy metabolism in T2DM and obesity. Mitochondrial dysfunction also promotes neuronal susceptibility to oxidative damage, and weakens synapses.

Various factors, including SCFAs, bile acids, inflammatory cytokines, and pathways of oxidation/antioxidants, can allow gut microbiota to impact mitochondria. For example, dysbiosis-induced inflammation can lead to a higher level of reactive oxygen species (ROS) in the body. Conversely, a decreased level of useful SCFAs may negatively affect how cells control the generation of energy. As it turns out, this feedback between the gut microbiota and the mitochondria of host cells is recognized today as a significant pathway in linking metabolic disturbances and neurodegeneration throughout the body [73,74]. Mitochondrial dysfunction may further amplify inflammatory signaling and oxidative stress, while altered microbial metabolites and chronic inflammation can worsen mitochondrial injury, thereby creating a self-perpetuating pathogenic cycle linking metabolic and neurodegenerative disorders [75].

### 6.5. Insulin Resistance and Neurodegeneration

Insulin resistance is not just limited to peripheral issues. Impaired brain insulin signaling is critical in supporting neuroprotection, neuronal survival, the plasticity of synapses, and memory functions, as well as energy expenditure and appetite regulation. Impaired brain insulin signaling has been associated with cognitive decline and Alzheimer’s-related neurodegenerative changes. Although some studies have described AD as exhibiting ‘brain insulin resistance’-like features, this concept remains a mechanistic hypothesis rather than a formal disease classification [76]. Altered gut microbiota also plays an insidious role in insulin resistance, driving it via an increase in inflammation and/or endotoxemia, altered metabolism of bile acids, and reduced levels of short-chain fatty acids (SCFAs). Changes may promote brain inflammation, neurodegeneration, and cognitive impairment. Impaired insulin receptor signaling pathways, particularly PI3K/Akt signaling, together with neuronal glucose transporter dysfunction and cerebral glucose hypometabolism, may further contribute to synaptic dysfunction and neurodegeneration. Insulin resistance has also been associated with tau hyperphosphorylation and progressive cognitive decline. Taken together, insulin resistance may represent one of several overlapping mechanisms linking T2DM, obesity, and Alzheimer’s disease. Gut–brain–immune interactions may contribute to overlapping inflammatory and metabolic features observed across metabolic and neurodegenerative disorders [76]. This is not just through one pathway but many. Dysbiosis has simultaneous impacts at multiple interlinked biological stages: at the immunological system level via inflammation and at the metabolic stage, involving altered nutrient and energy pathways, mitochondrial activity, and function, as well as neural transmission pathways and cognitive functions. Accordingly, studies need to use multiple omics analyses to concurrently capture all relevant microbial, metabolic, transcriptomic, and immunologic components.

Although multi-omics studies have identified numerous microbial, inflammatory, and metabolic signatures associated with neurodegenerative and metabolic diseases, only a limited number have undergone robust clinical validation for diagnostic or therapeutic applications.

## 7. Therapeutic and Translational Perspectives

Not only is the gut–brain–immune axis crucial for uncovering disease mechanisms, it also presents several possible therapeutic applications. Since many of its components are modifiable (e.g., diet, gut flora, inflammation, metabolism), modulating the gut–brain–immune axis may represent a promising therapeutic strategy; however, many proposed interventions remain investigational, and mechanistic as well as clinical evidence is still evolving. However, treatments based on these discoveries are mostly still in early development and will require extensive longitudinal clinical trials before implementation in routine clinical precision medicine practice [66].

### 7.1. Probiotics, Prebiotics, and Psychobiotics

Microbiome-based therapeutics include probiotics, which are live beneficial microorganisms such as *Bifidobacterium* and *Lactobacillus*, and prebiotics, which are dietary substrates that support the growth and activity of beneficial gut microbes [77]. More recently, a special class of psychobiotics has come into view, with claims ranging from improved depression, to better cognition, a healthier stress response, and less neuroinflammation. Psychobiotics are defined as probiotics or related microbial interventions that may beneficially influence mental health through gut–brain signaling pathways. Commonly investigated strains include *Lactobacillus* and *Bifidobacterium* species, which have been studied in relation to anxiety, depression, stress responses, and cognitive function. While preclinical studies have demonstrated promising neuroimmune and behavioral effects, clinical evidence in humans remains limited and sometimes inconsistent [78]. A review paper on psychobiotics demonstrated that psychobiotics can interact through metabolic outputs, vagal nerve signaling pathways, regulation of the immune system, and the hypothalamic–pituitary axis [79]. Though many clinical trial outcomes are ambiguous or inconsistent, a positive tendency was noted, and clinical investigations are called for with a focus on standardized formulations, optimized dose responses, and stratification of particular patient groups. However, the interpretation of probiotic and psychobiotic studies remains challenging because of substantial heterogeneity in microbial strains, dosing regimens, treatment duration, study populations, and clinical trial design, which may contribute to inconsistent reproducibility across studies.

### 7.2. Diet and Precision Nutrition

Diet is perhaps the most potent mediator of the human microbiome’s composition and function, influencing both diversity and metabolic potential. Studies suggest that high-fiber diets, plant-polyphenol-rich diets, and the Mediterranean diet increase microbial diversity and the production of salutary fermentation metabolites like butyrate. Additional dietary strategies including ketogenic diets, intermittent fasting, caloric restriction, and polyphenol-rich nutritional interventions have also been associated with modulation of gut microbiota composition, metabolic signaling, oxidative stress, and neuroinflammatory pathways [80]. Conversely, a Western-style diet—marked by an abundance of processed foods, high saturated fat, and a lack of fiber—promotes dysbiosis and the disruption of gut barrier integrity, leading to heightened gut permeability and metabolic inflammation. Precision nutrition—based on individual variability in microbiome structure, metabolic profile, genetic makeup, and daily lifestyle patterns—has rapidly emerged as a crucial translational strategy to address the broad spectrum of dietary responses across the general population and in individuals with obesity, T2DM, or cognitive decline, where responses to a particular diet might differ. Strategies employing multi-omics (integrating microbiome sequencing, metabolomics, genomics, and clinical data) are being developed to enable precision nutrition to personalize dietary recommendations tailored to individual profiles and predispositions [81]. However, precision nutrition approaches remain limited by substantial inter individual variability, lack of long-term validation studies, challenges in integrating complex multi-omics datasets, and difficulties in translating predictive models into routine clinical practice.

### 7.3. Fecal Microbiota Transplantation

Fecal microbiota transplantation (FMT) is utilized with the intention of restoring microbial diversity within a recipient by transferring the gut microbiome from a healthy volunteer donor. It has become the recognized standard of treatment in the event of recurrent *Clostridioides difficile* infection, but its therapeutic potential in chronic neurodegenerative as well as metabolic conditions still remains under investigation. Increasingly, research indicates that the gut bacteria and their metabolites may have the capacity to affect the brain directly, potentially impacting neuroinflammation, changes in microbiome metabolic profiles, the permeability of the gut barrier, and therefore the development or worsening of diseases involving communication between the gut and the brain [82]. Despite its exciting potential, a great deal of attention needs to be devoted to appropriate donor vetting, standardized methods of preparation, and storage and administration of microbiota, as well as to the long-term security concerns of FMT. The degree to which the process will be successful will probably also be affected by donor variability in terms of microbiome composition, the state of the recipient’s intestinal microbiome, the particular disease involved, and the delivery route used. At present, the use of FMT in neurodegenerative or metabolic diseases should be restricted to experimental or investigative efforts. Despite promising findings, FMT remains associated with important safety and translational concerns, including risks of pathogen transmission, immune-related complications, donor-selection variability, and ethical and regulatory challenges that currently limit widespread clinical application. Emerging next-generation microbiome therapeutics, including defined microbial consortia, engineered probiotics, postbiotics, and bacteriophage-based therapies, are also being explored as potentially safer and more targeted alternatives to conventional FMT [83].

### 7.4. Anti-Inflammatory and Metabolite-Based Strategies

**Figure 2:**
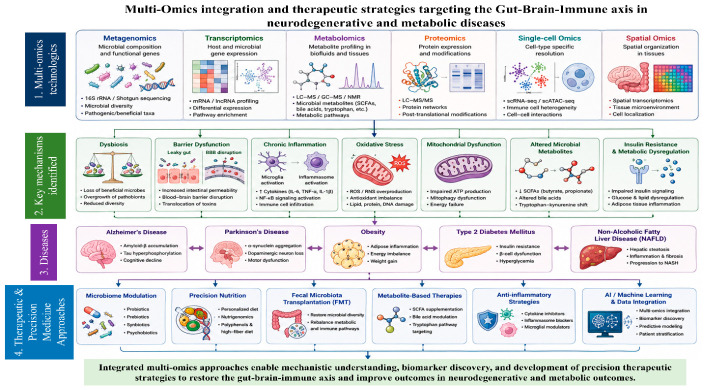
Multi-omics integration and therapeutic strategies targeting the gut–brain–immune axis.

Another therapeutic pathway in the gut–brain–immune nexus is the targeting of inflammation, as inflammation plays a critical role in initiating inflammatory signaling. Various cellular processes associated with this are targeted, including inflammasome activation, NF-κB signaling, glial inflammation (microglial inflammation), and the induction of inflammation caused by over-secretion of certain cytokines. Alternatively, individual gut microbiota metabolites have become candidates for novel treatments. SCFA supplementation, modifications of bile acid production, and targeted use of the tryptophan metabolic pathway have all demonstrated some efficacy in metabolic or neuroimmune regulation [62]. A limitation of metabolite-based approaches is the context-dependent activity of microbial metabolites. Many metabolites can be beneficial or detrimental depending on the target tissue or stage of the disease (Figure 2). Additional emerging therapeutic strategies include NLRP3 inflammasome inhibitors, GLP-1 receptor agonists, bile acid receptor modulators such as FXR and TGR5 agonists, and mitochondrial-targeted antioxidants aimed at reducing oxidative stress and neuroimmune dysfunction. Importantly, microbial metabolites may exert tissue-specific and concentration-dependent biological effects, making standardization of systemic metabolite-based therapeutic strategies challenging in clinical settings.

### 7.5. AI-Driven Precision Medicine

The findings of studies might support the clinical translation of brain–gut–immune axis findings through the combination of machine learning (ML) and the integration of large, multi-omics data (e.g., genomics, transcriptomics, metabolomics, proteomics, imaging) from different patient populations to discover meaningful signatures in diseases and their corresponding potential responders. The use of ML techniques to associate microbial functions and compositions with clinical phenotypes [47] aims at developing new approaches to personalize microbiome interventions as a form of precision medicine. Artificial intelligence could also improve early risk stratification for neurodegenerative or metabolic disorders, possibly prior to the occurrence of clinical symptoms. The application of ML would open opportunities for tailoring individualized probiotics, diets, or metabolites to these patients. Nonetheless, it is of great importance to ensure solid validation of these approaches on large diverse populations, since the microbiome composition can change with geographical place, diet, medication, age and the state of a disease. Overall, treatments of the brain–gut–immune axis are encouraging and represent a developing field with potential [50]. From a practical point of view, likely, short-term benefits will emerge from personalized nutrition plans, microbiome risk assessment, or adjuvant use of probiotics and prebiotics, whereas longer-term therapies including FMT, engineered probiotics, and novel therapies based on microbial metabolites still need to show solid proof in large-scale clinical trials before being applied broadly in clinical practice. However, important challenges remain, including a lack of standardized datasets, reproducibility concerns, model overfitting, limited external validation, and ethical and privacy issues associated with the integration of large-scale clinical and multi-omics patient data. Although AI-based approaches show considerable promise, many current machine learning models remain exploratory and require prospective validation in large, multi-center clinical cohorts before routine clinical implementation [77].

Despite significant advances in gut–brain–immune axis research, important translational barriers remain. These include substantial microbiome variability across populations, a lack of standardized multi-omics analytical pipelines, limited reproducibility between studies, difficulties in patient stratification, and regulatory challenges associated with microbiome-based therapeutics and large-scale clinical data integration. Addressing these limitations will be essential for successful clinical translation and precision medicine implementation [84].

Future personalized medicine strategies may increasingly integrate microbiome composition, host genetic susceptibility, immune phenotyping, metabolic profiling, and lifestyle/environmental factors to improve disease stratification and optimize targeted therapeutic interventions.

Additional ethical and regulatory considerations related to AI-driven medicine, microbiome engineering, personalized omics profiling, and fecal microbiota transplantation will also require careful attention, particularly regarding patient privacy, long-term safety, informed consent, data governance, and equitable clinical implementation [81].

## 8. Conclusions and Future Directions

The gut–brain–immune axis is a major regulatory network, coupling intestinal bacteria with immune system signaling, metabolism, and brain function. Accumulating evidence from multi-omics and clinical studies suggests strong associations between gut microbial dysbiosis and neurodegenerative (AD/PD) as well as metabolic diseases (obesity, T2DM), although many mechanistic relationships remain incompletely understood. Rather than treating Alzheimer’s disease, Parkinson’s disease, obesity, and T2DM as entirely distinct entities, these disorders may share overlapping inflammatory, metabolic, and neuroimmune mechanisms within the gut–brain–immune axis. Additional overlapping mechanisms including NLRP3 inflammasome activation, impaired autophagy, mitochondrial quality-control dysfunction, and altered insulin signaling pathways may further contribute to both metabolic and neurodegenerative disease progression [85]. New multi-omics techniques (metagenomics, transcriptomics, metabolomics, proteomics, single-cell seq, etc.) are greatly expanding knowledge of these complex biological networks and allow for their integration to detect disease-associated bacterial, immunological, and metabolic features, which have great potential for diagnosis and treatment. There are now advanced AI approaches being employed to process and interpret such massive omics data and are paving the path towards precision medicine. Challenges include variation in study outcomes because of differences in geography, diet, genetics, medication, sequencing technology, bioinformatics pipeline, and so on [86]. Additional limitations include small cohort sizes, batch effects, poor reproducibility across studies, heterogeneity in bioinformatic analyses, and the limited ability of many current studies to establish causal relationships. Most current studies are cross-sectional, which makes it impossible to determine cause and effect. And we do not have a complete understanding of the significance of some changes. So, standardization of multi-omics and longitudinal follow-ups will be essential. Integrated systems biology approaches combining microbiome, immune, metabolic, transcriptomic, proteomic, and clinical data through standardized analytical pipelines and multimodal longitudinal cohort studies will be critical for improving mechanistic understanding and clinical translation. Future efforts must include the integration of gut microbiome analyses with host immune and metabolic features in multi-dimensional approaches and use diverse cohorts, single-cell technology, AI, and other techniques to expand knowledge of the cell-specific and tissue-specific effects in this axis. Treatments might include personalized probiotics, prebiotics, and metabolites and personalized microbiome manipulation in humans. The gut–brain–immune axis is likely to be one of the quickest-evolving fields of medical translational research. The integration of omics with basic and clinical studies may lead to the personalized treatment of metabolic disorders and brain dysfunction together. However, most microbiome-based and precision medicine interventions remain investigational, and large-scale longitudinal multi-center validation studies are still required before widespread clinical implementation. A major strength of this review is the integration of multi-omics and AI-driven analytical perspectives across both neurodegenerative and metabolic diseases within a unified gut–brain–immune framework [87]. Future research should also prioritize causal mechanistic studies, validation in large and diverse human cohorts, and well-designed interventional clinical trials evaluating microbiome-targeted therapies and precision medicine approaches. Future studies should also consider the influence of host genetics, environmental exposures, circadian rhythm disruption, aging, diet, and lifestyle-related factors as important modulators of gut–brain–immune interactions and disease susceptibility.

Advancing this rapidly evolving field will require close interdisciplinary collaboration among microbiology, neuroscience, immunology, metabolomics, computational biology, bioinformatics, and clinical medicine to successfully translate multi-omics discoveries into effective precision diagnostics and therapeutics.

## Figures and Tables

**Table 1:** Overview of major multi-omics technologies applied in gut–brain–immune axis research.

Technology	Biological Material Analyzed	Major Applications in Gut–Brain–Immune Axis Research	Strengths	Limitations
**Metagenomics**	Stool samples, intestinal biopsies	Characterization of gut microbial diversity, functional microbial genes, dysbiosis profiling in AD, PD, obesity, T2DM	Comprehensive microbial profiling; identifies taxonomic and functional composition	Limited species-level resolution in 16S sequencing; batch effects; cannot distinguish viable/non-viable microbes
**Transcriptomics**	Brain tissue, intestinal tissue, immune cells, blood	Analysis of host inflammatory signaling, immune activation, mitochondrial dysfunction, neuroimmune pathways	Identifies disease-associated gene-expression changes	Bulk transcriptomics lacks cell-specific resolution
**Metatranscriptomics**	Microbial RNA from stool or tissue samples	Evaluation of actively expressed microbial genes and metabolic activity	Captures functional microbial activity	Technically complex; RNA instability; computationally intensive
**Metabolomics**	Serum, plasma, CSF, stool, urine	Analysis of SCFAs, bile acids, tryptophan metabolites, TMAO, oxidative stress metabolites	Reflects functional metabolic interactions between host and microbiota	Metabolite levels influenced by diet, medications, and environment
**Proteomics**	Plasma, CSF, brain tissue, intestinal tissue	Identification of inflammatory cytokines, signaling proteins, oxidative stress markers	Direct assessment of functional protein networks	Protein complexity and variability complicate interpretation
**Single-Cell Omics**	Individual immune cells, neurons, glial cells, intestinal epithelial cells	Characterization of cellular heterogeneity and cell-specific inflammatory responses	High cellular resolution; identifies rare cell populations	High cost; computational complexity
**Spatial Omics**	Preserved tissue sections	Spatial mapping of neuroimmune interactions and tissue-specific signaling	Preserves tissue architecture and spatial relationships	Tissue preservation challenges; limited spatial resolution
**AI/Machine Learning Integration**	Integrated multi-omics and clinical datasets	Biomarker discovery, predictive modeling, patient stratification, precision medicine	Detects complex nonlinear biological relationships	Risk of overfitting; limited external validation; interpretability and ethical concerns

**Table 2:** Comparative summary of gut–brain–immune alterations across neurological and neuropsychiatric disorders.

Disease	Microbiota Alterations	Key Inflammatory Pathways	Implicated Metabolites	Major Omics Findings
Alzheimer’s disease	Reduced microbial diversity; reduced SCFA-producing bacteria	Microglial activation, IL-1β, IL-6, TNF-α, NLRP3 inflammasome	Reduced SCFAs, altered bile acids, tryptophan metabolites	Metagenomics and metabolomics link dysbiosis with amyloid pathology and cognitive decline
Parkinson’s disease	Reduced beneficial SCFA-producing taxa; increased pro-inflammatory taxa	NF-κB activation, oxidative stress, intestinal inflammation	Reduced SCFAs, altered dopamine-related metabolites	Multi-omics links dysbiosis with α-synuclein aggregation, mitochondrial dysfunction, and gut-origin mechanisms
Multiple sclerosis	Increased pro-inflammatory taxa; reduced anti-inflammatory bacteria	Th17/Treg imbalance, BBB disruption, autoimmune inflammation	SCFAs, tryptophan-related metabolites	Transcriptomics and single-cell omics identify immunecell heterogeneity and neuroinflammatory signatures
Depression/neuropsychiatric disorders	Altered microbial diversity; inconsistent cohort-specific signatures	HPAaxis dysregulation, chronic inflammation, cytokine signaling	Tryptophan–kynurenine metabolites, serotonin-related metabolites	Metagenomics and metabolomics associate dysbiosis with mood regulation and neuroinflammation

**Table 3:** Multi-omics findings linking the gut–brain–immune axis with neurodegenerative and metabolic diseases.

Disease	Major Gut Microbiota Alterations	Key Immune/Inflammatory Mechanisms	Important Microbial Metabolites	Major Multi-Omics Findings	Potential Therapeutic Implications	Key References
**Alzheimer’s disease (AD)**	Reduced microbial diversity; decreased SCFA-producing bacteria	Microglial activation; IL-1β, IL-6, TNF-α; neuroinflammation	Reduced butyrate; altered bile acids; tryptophan metabolites	Metagenomics and metabolomics associated dysbiosis with amyloid pathology and cognitive decline	Probiotics, SCFA modulation, anti-inflammatory targeting	[39,41]
**Parkinson’s disease (PD)**	Reduced beneficial SCFA-producing taxa; increased pro-inflammatory microbes	NF-κB activation; oxidative stress; intestinal inflammation	Reduced SCFAs; altered dopamine-related metabolites	Experimental and clinical multi-omics studies linked with α-synuclein aggregation and mitochondrial dysfunction	Microbiome-targeted therapy; gut-origin interventions	[4,45]
**Multiple sclerosis (MS)**	Increased pro-inflammatory taxa; reduced anti-inflammatory bacteria	Th17/Treg imbalance; immune dysregulation; BBB dysfunction	SCFAs; tryptophan metabolites	Single-cell and transcriptomic analyses identified immunecell heterogeneity	Immune modulation; microbiome-based therapies	[49]
**Depression and neuropsychiatric disorders**	Altered microbial diversity and serotonin-associated bacteria	HPAaxis dysregulation; chronic inflammation	Tryptophan–kynurenine metabolites; serotonin-related metabolites	Metabolomics and metagenomics associated dysbiosis with mood disorders	Psychobiotics; precision psychiatry	[51]
**Obesity**	Reduced microbial diversity; altered Firmicutes/Bacteroidetes balance	Chronic low-grade inflammation; metabolic endotoxemia	SCFAs; bile acid derivatives	Multi-omics studies linked dysbiosis with adipose inflammation and insulin resistance	Dietary interventions; microbiome modulation	[53,54]
**Type 2 diabetes mellitus (T2DM)**	Reduced butyrate-producing bacteria; increased opportunistic pathogens	Inflammasome activation; chronic cytokine signaling	Reduced SCFAs; altered BCAAs and bile acids	Integrated microbiome–metabolome analyses identified metabolic dysfunction signatures	Precision metabolic medicine; GLP-1-related interventions	[56]
**Metabolic dysfunction-associated steatotic liver disease (MASLD/MASH)**	Dysbiosis-associated intestinal permeability	Hepatic inflammation; oxidative stress; Kupffer-cell activation	Altered bile acids; choline metabolites; SCFAs	Metabolomic and microbiome studies linked dysbiosis with steatosis and fibrosis	Microbiome-directed liver therapies	[59]
**Shared mechanisms across disorders**	Dysbiosis and reduced beneficial metabolites	NF-κB signaling; oxidative stress; mitochondrial dysfunction; metaflammation	SCFAs; LPS; tryptophan metabolites	Multi-omics integration identified overlapping inflammatory and metabolic pathways	Systems-level and precision therapeutic strategies	[62,63]

## Data Availability

No new data were created or analyzed in this study.

## References

[1] Cryan J.F., O’Riordan K.J., Cowan C.S., Sandhu K.V., Bastiaanssen T.F., Boehme M., Codagnone M.G., Cussotto S., Fulling C., Golubeva A.V. (2019). The microbiota–gut–brain axis. Physiol. Rev..

[2] Fan Y., Pedersen O. (2021). Gut microbiota in human metabolic health and disease. Nat. Rev. Microbiol..

[3] Fung T.C., Olson C.A., Hsiao E.Y. (2017). Interactions between the microbiota, immune and nervous systems in health and disease. Nat. Neurosci..

[4] Houser M.C., Tansey M.G. (2017). The gut–brain axis: Is intestinal inflammation a silent driver of Parkinson’s disease pathogenesis?. npj Park. Dis..

[5] Sharon G., Sampson T.R., Geschwind D.H., Mazmanian S.K. (2016). The central nervous system and the gut microbiome. Cell.

[6] Galicia-Garcia U., Benito-Vicente A., Jebari S., Larrea-Sebal A., Siddiqi H., Uribe K.B., Ostolaza H., Martín C. (2020). Pathophysiology of type 2 diabetes mellitus. Int. J. Mol. Sci..

[7] Del Cornò M., Aureli A., Varano B., Conti L. (2026). Endotoxins and Metabolic Endotoxemia in Obesity and Associated Noncommunicable Diseases: A Focus on Sex Differences. Biomolecules.

[8] Baars A., Oosting A., Knol J., Garssen J., Van Bergenhenegouwen J. (2015). The gut microbiota as a therapeutic target in IBD and metabolic disease: A role for the bile acid receptors FXR and TGR5. Microorganisms.

[9] Tilg H., Zmora N., Adolph T.E., Elinav E. (2020). The intestinal microbiota fuelling metabolic inflammation. Nat. Rev. Immunol..

[10] Hasin Y., Seldin M., Lusis A. (2017). Multi-omics approaches to disease. Genome Biol..

[11] Chen Y., Xu J., Chen Y. (2021). Regulation of neurotransmitters by the gut microbiota and effects on cognition in neurological disorders. Nutrients.

[12] Lynch S.V., Pedersen O. (2016). The human intestinal microbiome in health and disease. N. Engl. J. Med..

[13] Silva Y.P., Bernardi A., Frozza R.L. (2020). The role of short-chain fatty acids from gut microbiota in gut–brain communication. Front. Endocrinol..

[14] Chen H., Yu S., Zhang M., Tian B., Yang L., Lu J. (2026). Gut microbial metabolites in inflammatory bowel disease: Immunological mechanisms regulating Treg/Th17 balance and therapeutic potential. Front. Immunol..

[15] Breit S., Kupferberg A., Rogler G., Hasler G. (2018). Vagus nerve as modulator of the brain–gut axis in psychiatric and inflammatory disorders. Front. Psychiatry.

[16] Rusch J.A., Layden B.T., Dugas L.R. (2023). Signalling cognition: The gut microbiota and hypothalamic-pituitary-adrenal axis. Front. Endocrinol..

[17] Dehhaghi M., Kazemi Shariat Panahi H., Guillemin G.J. (2019). Microorganisms, tryptophan metabolism, and kynurenine pathway: A complex interconnected loop influencing human health status. Int. J. Tryptophan Res..

[18] Wiertsema S.P., van Bergenhenegouwen J., Garssen J., Knippels L.M. (2021). The interplay between the gut microbiome and the immune system in the context of infectious diseases throughout life and the role of nutrition in optimizing treatment strategies. Nutrients.

[19] Dantzer R. (2018). Neuroimmune interactions: From the brain to the immune system and vice versa. Physiol. Rev..

[20] Cekanaviciute E., Buckwalter M.S. (2016). Astrocytes: Integrative regulators of neuroinflammation in stroke and other neurological diseases. Neurotherapeutics.

[21] Yu T., Wang Z., Chen Y., Xiang Y., Wu M., Zhang M., Yin X., Chen Z. (2025). Blood–brain barrier (BBB) dysfunction in CNS diseases: Paying attention to pericytes. CNS Neurosci. Ther..

[22] Parker A., Fonseca S., Carding S.R. (2020). Gut microbes and metabolites as modulators of blood–brain barrier integrity and brain health. Gut Microbes.

[23] de Souza P.A., Ramos J.N., Vasconcellos L., Costa L.V., Forsythe S.J., Brandão M.L. (2025). Application and Limitations of 16S rRNA Gene Sequencing for Identifying WHO Priority Pathogenic Gram-Negative Bacilli. Infect. Drug Resist..

[24] Gilbert J.A., Blaser M.J., Caporaso J.G., Jansson J.K., Lynch S.V., Knight R. (2018). Current understanding of the human microbiome. Nat. Med..

[25] Shen Y., Fan N., Ma S.X., Cheng X., Yang X., Wang G. (2025). Gut microbiota dysbiosis: Pathogenesis, diseases, prevention, and therapy. MedComm.

[26] Jain V., Baraniya D., El-Hadedy D.E., Chen T., Slifker M., Alakwaa F., Cai K.Q., Chitrala K.N., Fundakowski C., Al-Hebshi N.N. (2023). Integrative metatranscriptomic analysis reveals disease-specific microbiome–host interactions in oral squamous cell carcinoma. Cancer Res. Commun..

[27] Chen S., Zhou Y., Chen Y., Gu J. (2018). fastp: An ultra-fast all-in-one FASTQ preprocessor. Bioinformatics.

[28] Sarapultsev A., Gusev E., Komelkova M., Utepova I., Luo S., Hu D. (2023). JAK-STAT signaling in inflammation and stress-related diseases: Implications for therapeutic interventions. Mol. Biomed..

[29] Needham B.D., Funabashi M., Adame M.D., Wang Z., Boktor J.C., Haney J., Wu W.L., Rabut C., Ladinsky M.S., Hwang S.J. (2022). A gut-derived metabolite alters brain activity and anxiety behaviour in mice. Nature.

[30] Chiurchiù V., Tiberi M., Matteocci A., Fazio F., Siffeti H., Saracini S., Mercuri N.B., Sancesario G. (2022). Lipidomics of bioactive lipids in Alzheimer’s and Parkinson’s diseases: Where are we?. Int. J. Mol. Sci..

[31] Gao K., Mu C.L., Farzi A., Zhu W.Y. (2020). Tryptophan metabolism: A link between the gut microbiota and brain. Adv. Nutr..

[32] Xiong L.L., Xue L.L., Chen Y.J., Du R.L., Wang Q., Wen S., Zhou L., Liu T., Wang T.H., Yu C.Y. (2021). Proteomics study on the cerebrospinal fluid of patients with encephalitis. ACS Omega.

[33] Johnson E.C., Dammer E.B., Duong D.M., Ping L., Zhou M., Yin L., Higginbotham L.A., Guajardo A., White B., Troncoso J.C. (2020). Large-scale proteomic analysis of Alzheimer’s disease brain and cerebrospinal fluid reveals early changes in energy metabolism associated with microglia and astrocyte activation. Nat. Med..

[34] Morais L.H., Schreiber H.L., Mazmanian S.K. (2021). The gut microbiota–brain axis in behaviour and brain disorders. Nat. Rev. Microbiol..

[35] Zhou R., Yang G., Zhang Y., Wang Y. (2023). Spatial transcriptomics in development and disease. Mol. Biomed..

[36] Pammi M., Aghaeepour N., Neu J. (2023). Multi-omics, artificial intelligence, and precision medicine in perinatology. Pediatr. Res..

[37] Sharma A., Lysenko A., Jia S., Boroevich K.A., Tsunoda T. (2024). Advances in AI and machine learning for predictive medicine. J. Hum. Genet..

[38] Tanaka M. (2025). From serendipity to precision: Integrating AI, multi-omics, and human-specific models for personalized neuropsychiatric care. Biomedicines.

[39] Vogt N.M., Kerby R.L., Dill-McFarland K.A., Harding S.J., Merluzzi A.P., Johnson S.C., Carlsson C.M., Asthana S., Zetterberg H., Blennow K. (2017). Gut microbiome alterations in Alzheimer’s disease. Sci. Rep..

[40] Di Vincenzo F., Del Gaudio A., Petito V., Lopetuso L.R., Scaldaferri F. (2024). Gut microbiota, intestinal permeability, and systemic inflammation: A narrative review. Intern. Emerg. Med..

[41] Heneka M.T., Carson M.J., El Khoury J., Landreth G.E., Brosseron F., Feinstein D.L., Jacobs A.H., Wyss-Coray T., Vitorica J., Ransohoff R.M. (2015). Neuroinflammation in Alzheimer’s disease. Lancet Neurol..

[42] Sampson T. (2025). Microbial amyloids in neurodegenerative amyloid diseases. FEBS J..

[43] Costa H.N., Esteves A.R., Empadinhas N., Cardoso S.M. (2023). Parkinson’s disease: A multisystem disorder. Neurosci. Bull..

[44] Rietdijk C.D., Perez-Pardo P., Garssen J., Van Wezel R.J., Kraneveld A.D. (2017). Exploring Braak’s hypothesis of Parkinson’s disease. Front. Neurol..

[45] Lubomski M., Tan A.H., Lim S.Y., Holmes A.J., Davis R.L., Sue C.M. (2020). Parkinson’s disease and the gastrointestinal microbiome. J. Neurol..

[46] Shen L., Dettmer U. (2024). Alpha-synuclein effects on mitochondrial quality control in parkinson’s disease. Biomolecules.

[47] Taj T., Kaushik M., Islam A., Das J., Kumar B., Hussain M.S., Ramzan M., Ashique S., Tariq M., Sridhar S.B. (2025). Microbiota-brain interaction: The role of gut-derived proteins in addressing various neurological disorders including Parkinson’s (PD) and Alzheimer’s diseases (AD). Biomed. Pharmacother..

[48] Chu F., Shi M., Lang Y., Shen D., Jin T., Zhu J., Cui L. (2018). Gut microbiota in multiple sclerosis and experimental autoimmune encephalomyelitis: Current applications and future perspectives. Mediat. Inflamm..

[49] Cekanaviciute E., Yoo B.B., Runia T.F., Debelius J.W., Singh S., Nelson C.A., Kanner R., Bencosme Y., Lee Y.K., Hauser S.L. (2017). Gut bacteria from multiple sclerosis patients modulate human T cells and exacerbate symptoms in mouse models. Proc. Natl. Acad. Sci. USA.

[50] Ren J., Niu Z., Wang J., Guo J., Hao H., Gao F., Liu R., Wang Z. (2025). The link between gut microbiota and multiple sclerosis from the perspective of barrier function. Front. Immunol..

[51] Clapp M., Aurora N., Herrera L., Bhatia M., Wilen E., Wakefield S. (2017). Gut microbiota’s effect on mental health: The gut–brain axis. Clin. Pract..

[52] Abenavoli L., Scarlata G.G., Scarpellini E., Boccuto L., Spagnuolo R., Tilocca B., Roncada P., Luzza F. (2023). Metabolic-dysfunction-associated fatty liver disease and gut microbiota: From fatty liver to dysmetabolic syndrome. Medicina.

[53] Turnbaugh P.J., Ley R.E., Mahowald M.A., Magrini V., Mardis E.R., Gordon J.I. (2006). An obesity-associated gut microbiome with increased capacity for energy harvest. Nature.

[54] Cani P.D., Amar J., Iglesias M.A., Poggi M., Knauf C., Bastelica D., Neyrinck A.M., Fava F., Tuohy K.M., Chabo C. (2007). Metabolic endotoxemia initiates obesity and insulin resistance. Diabetes.

[55] Guria S., Hoory A., Das S., Chattopadhyay D., Mukherjee S. (2023). Adipose tissue macrophages and their role in obesity-associated insulin resistance: An overview of the complex dynamics at play. Biosci. Rep..

[56] Qin J., Li Y., Cai Z., Li S., Zhu J., Zhang F., Liang S., Zhang W., Guan Y., Shen D. (2012). A metagenome-wide association study of gut microbiota in type 2 diabetes. Nature.

[57] Jin J.Y., Yang X.Y., Feng R., Ye M.L., Xu H., Wang J.Y., Hu J.C., Zuo H.T., Lu J.Y., Song J.Y. (2025). Gut Microbiota-Derived Metabolites Orchestrate Metabolic Reprogramming in Diabetic Cardiomyopathy: Mechanisms and Therapeutic Frontiers. FASEB J..

[58] Mei J., Yang F.Y., Gong Q. (2026). Branched-chain amino acids and insulin resistance in type 2 diabetes: From metabolic dysregulation to therapeutic targets. Front. Endocrinol..

[59] Tilg H., Moschen A.R. (2014). Microbiota and diabetes: An evolving relationship. Gut.

[60] Qin P., Sun Y., Li L. (2024). Mitochondrial dysfunction in chronic neuroinflammatory diseases. Int. J. Mol. Med..

[61] Arreguín-Cano J.A., Santana-Delgado S.A., Villegas-Mercado C.E., Orozco-Molina G.G., González-Acosta A., Bermúdez M. (2026). Linking inflammation, metabolic dysfunction, and neurodegeneration: A comprehensive review of TLR2 pathways in type 2 diabetes. Front. Clin. Diabetes Healthc..

[62] Loh J.S., Mak W.Q., Tan L.K., Ng C.X., Chan H.H., Yeow S.H., Foo J.B., Ong Y.S., How C.W., Khaw K.Y. (2024). Microbiota–gut–brain axis and its therapeutic applications in neurodegenerative diseases. Signal Transduct. Target. Ther..

[63] Munir M.U., Ali S.A., Chung K.H., Kakinen A., Javed I., Davis T.P. (2024). Reverse engineering the gut–brain axis and microbiome-metabolomics for symbiotic/pathogenic balance in neurodegenerative diseases. Gut Microbes.

[64] Khan M.S., Hegde V. (2020). Obesity and diabetes mediated chronic inflammation: A potential biomarker in Alzheimer’s disease. J. Pers. Med..

[65] Han Q., Li W., Chen P., Wang L., Bao X., Huang R., Liu G., Chen X. (2024). Microglial NLRP3 inflammasome-mediated neuroinflammation and therapeutic strategies in depression. Neural Regen. Res..

[66] O’Riordan K.J., Moloney G.M., Keane L., Clarke G., Cryan J.F. (2025). The gut microbiota–immune–brain axis: Therapeutic implications. Cell Rep. Med..

[67] Ponce-Lopez T. (2025). Peripheral inflammation and insulin resistance: Their impact on blood–brain barrier integrity and glia activation in Alzheimer’s disease. Int. J. Mol. Sci..

[68] Tang W., Zhu H., Feng Y., Guo R., Wan D. (2020). The impact of gut microbiota disorders on the blood–brain barrier. Infect. Drug Resist..

[69] Dithmer S., Blasig I.E., Fraser P.A., Qin Z., Haseloff R.F. (2024). The basic requirement of tight junction proteins in blood-brain barrier function and their role in pathologies. Int. J. Mol. Sci..

[70] Wang Y., Zhang Y., Wang W., Zhang Y., Dong X., Liu Y. (2025). Diverse physiological roles of kynurenine pathway metabolites: Updated implications for health and disease. Metabolites.

[71] Jung Y.H., Chae C.W., Han H.J. (2024). The potential role of gut microbiota-derived metabolites as regulators of metabolic syndrome-associated mitochondrial and endolysosomal dysfunction in Alzheimer’s disease. Exp. Mol. Med..

[72] Zong Y., Li H., Liao P., Chen L., Pan Y., Zheng Y., Zhang C., Liu D., Zheng M., Gao J. (2024). Mitochondrial dysfunction: Mechanisms and advances in therapy. Signal Transduct. Target. Ther..

[73] Morais L.H., Stiles L., Freeman M., Oguienko A.D., Hoang J.D., Ji J., Jones J., Quan B., Devine J., Bois J.S. (2025). The gut microbiome promotes mitochondrial respiration in the brain of a Parkinson’s disease mouse model. npj Park. Dis..

[74] Ju T., Zhang Y., Liu L., Zhao X., Li X., Liu C., Sun S., Wu L.A. (2026). The role of gut microbiota–mitochondria crosstalk in neurodegeneration: Underlying mechanisms and potential therapies. Neural Regen. Res..

[75] Kim M.E., Lim Y., Lee J.S. (2025). Mitochondrial dysfunction and metabolic reprogramming in chronic inflammatory diseases: Molecular insights and therapeutic opportunities. Curr. Issues Mol. Biol..

[76] Pliszka M., Szablewski L. (2026). Insulin Signaling in Alzheimer’s Disease: Association with Brain Insulin Resistance. Int. J. Mol. Sci..

[77] Ji J., Jin W., Liu S.J., Jiao Z., Li X. (2023). Probiotics, prebiotics, and postbiotics in health and disease. MedComm.

[78] Del Toro-Barbosa M., Hurtado-Romero A., Garcia-Amezquita L.E., García-Cayuela T. (2020). Psychobiotics: Mechanisms of action, evaluation methods and effectiveness in applications with food products. Nutrients.

[79] Ķimse L., Reinis A., Miķelsone-Jansone L., Gintere S., Krūmiņa A. (2024). A narrative review of psychobiotics: Probiotics that influence the gut–brain axis. Medicina.

[80] Dai H., Yang H., Wang R., Wang X., Zhang X. (2025). Modulating Gut Microbiota with Dietary Components: A Novel Strategy for Cancer–Depression Comorbidity Management. Nutrients.

[81] Nourazarain A., Vaziri Y. (2025). Nutrigenomics meets multi-omics: Integrating genetic, metabolic, and microbiome data for personalized nutrition strategies. Genes Nutr..

[82] Eslami M., Adampour Z., Fadaee Dowlat B., Yaghmayee S., Motallebi Tabaei F., Oksenych V., Naderian R. (2025). A novel frontier in gut–brain axis research: The transplantation of fecal microbiota in neurodegenerative disorders. Biomedicines.

[83] Ramesh A., Subbarayan R., Shrestha R., Adtani P.N. (2026). Exploring Fecal Microbiota Transplantation: Potential Benefits, Associated Risks, and Challenges in Cancer Treatment. Cancer Rep..

[84] Li Z., Samui S., Liu J.A., Yang Y., Liu X., Chen Q., Li J., Gopinath D., Luo P., Shan D. (2026). Gut microbiome and metabolic health: Mechanisms and precision interventions. Gut Microbes.

[85] Chen C., Wang G.Q., Li D.D., Zhang F. (2025). Microbiota–gut–brain axis in neurodegenerative diseases: Molecular mechanisms and therapeutic targets. Mol. Biomed..

[86] Duan D., Wang M., Han J., Li M., Wang Z., Zhou S., Xin W., Li X. (2025). Advances in multi-omics integrated analysis methods based on the gut microbiome and their applications. Front. Microbiol..

[87] Hemmati M.A., Monemi M., Asli S., Mohammadi S., Foroozanmehr B., Haghmorad D., Oksenych V., Eslami M. (2024). Using new technologies to analyze gut microbiota and predict cancer risk. Cells.

